# *Arabidopsis FHY3/CPD45* regulates far-red light signaling and chloroplast division in parallel

**DOI:** 10.1038/srep09612

**Published:** 2015-04-15

**Authors:** Ning Chang, Yuefang Gao, Lin Zhao, Xiaomin Liu, Hongbo Gao

**Affiliations:** 1 College of Biological Sciences and Biotechnology, Beijing Forestry University, Beijing, 100083, China

## Abstract

*CPD45* (*chloroplast division45*),which is also known as *FHY3* (*far-red elongated hypocotyl3*), is a key factor in the far-red light signaling pathway in *Arabidopsis*. We previously showed that *FHY3/CPD45* also regulates chloroplast division. Because light is also a regulator of chloroplast development and division, we sought to clarify the relationship between far-red light signaling and chloroplast division pathways. We found that the chloroplast division mutant *arc5-3* had no defect in far-red light sensing, and that constitutive overexpression of *ARC5* rescued the chloroplast division defect, but not the defect in far-red light signaling, of *cpd45*. *fhy1*, which is defective in far-red light signaling, exhibited normal chloroplast division. Constitutive overexpression of *FHY1* rescued the far-red light signaling defect, but not the chloroplast division defect, of *cpd45*. Moreover, *ARC5* and *FHY1* expression were not affected in *fhy1* and *arc5-3*, respectively. Based on these results, we propose that *FHY3/CPD45* regulates far-red light signaling and chloroplast division in parallel by activating the expression of *FHY1* and *ARC5* independently. This work demonstrates how relationships between different pathways in a gene regulatory network can be explored.

Plants adapt their growth according to changing environmental conditions, and one of the most important environmental inputs is light. Light not only provides energy for plants via photosynthesis, but also functions as a signal that regulates plant growth and development. Five types of light receptors have been reported in plants, namely phytochromes (phy)^1^, cryptochromes^2^, phototropins^3^, Zeitlupe^4^ and UVR8^5^,^6^. Five phys species exist in *Arabidopsis thaliana*, phyA to E^1^,^7^. PhyA, a red light (620 ~ 700 nm) and far-red light (700 ~ 800 nm)^8^ sensor, is imported into the nucleus upon light illumination, with the help of FHY1 (Far-red elongated Hypocotyl 1)^9^,^10^,^11^ and its homolog FHL (FHY1-like)^12^. FAR1 (far-red-impaired response)^13^ and FHY3 (also known as CPD45) are transcription factors in the same family that are involved in far-red light signaling through activating the expression of *FHY1* and *FHL*^14^. Due to their defects in far-red light signaling, *fhy3*, *fhy1*, and *phyA* plants have elongated hypocotyls under far-red light^15^,^16^,^17^.

Chloroplasts, which evolved from endosymbiotic cyanobacteria, are not only the major site of photosynthesis, but also the major site for numerous important biosynthetic pathways, such as fatty acid biosynthesis^18^, amino acid biosynthesis^19^, and the immune response in plants^20^. Chloroplasts are generated through the binary fission of preexisting chloroplasts^21^. In mutants with chloroplast division defects, chloroplasts are generally enlarged in size and reduced in number. The large chloroplasts are readily injured by high light intensities, due to their low mobility, which also affects photosynthesis and other metabolic pathways^22^,^23^.

Many genes involved in chloroplast division have been identified. For instance, *ARC5*, which is a nuclear gene, encodes a dynamin-related protein that is part of the chloroplast division machinery^24^. The *arc5* mutant has enlarged and dumbbell-shaped chloroplasts. Subsequently, mutants with a similar phenotype, *cpd45* and *cpd25*, were identified^25^. Map-based cloning revealed that *CPD45* was the same gene as *FHY3* and *CPD25* was the same gene as *FRS4*, a homolog of *CPD45*/*FHY3*^25^. Further analysis suggested that CPD45 worked cooperatively with CPD25 to activate the expression *ARC5*. Unlike *cpd45*, the hypocotyl length of *cpd25* was similar to that of the wild type under far-red light, suggesting that *CPD25* is not involved in far-red light signaling^25^.

Light affects many aspects of chloroplast development and function, including biogenesis, morphogenesis, movement, and biochemical metabolism^26^. Studies conducted in our lab and others suggest that *FHY3/CPD45* not only promotes chloroplast division by activating the expression of *ARC5*, but is also involved in light sensing by promoting the nuclear import of phyA, which is essential for its function in light signaling^16^,^25^,^27^,^28^. However, it is unclear whether *FHY3/CPD45* promotes chloroplast division through *FHY1* and *PHYA*. We addressed this question using a set of genetic experiments with related mutants and transgenic plants. Our analysis suggests that *FHY3/CPD45* regulates chloroplast division and far-red light signaling by activating the expression of *ARC5* and *FHY1* in parallel.

## Results

### Constitutive overexpression of *FHY1* rescued the far-red light sensing defect, but not the chloroplast division defect, of *cpd45*

**Figure 1:**
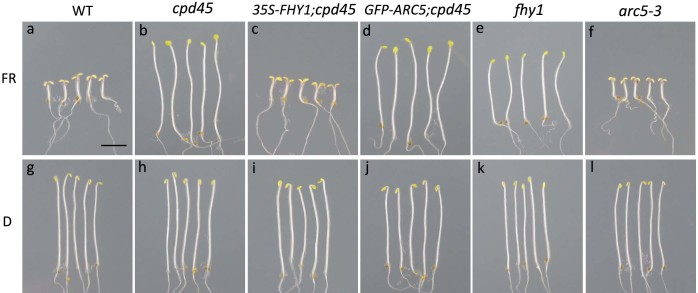
Seedlings of various genetic backgrounds grown in far-red light or the dark for 4 days. (a, g) Wild type. (b, h) *cpd45* mutant. (c, i) *cpd45* mutant transformed with the *35S-FHY1* transgene. (d, j) *cpd45* mutant transformed with the *35S-GFP-ARC5* transgene. (e, k) *fhy1* mutant. (f, l) *arc5-3* mutant. (a–f) seedlings grown in 10 μmol·m^−2^·s^−1^ far-red light, (g–l) seedings grown in the dark. WT, wild type. Scale bar represents 3 mm.

**Figure 2:**
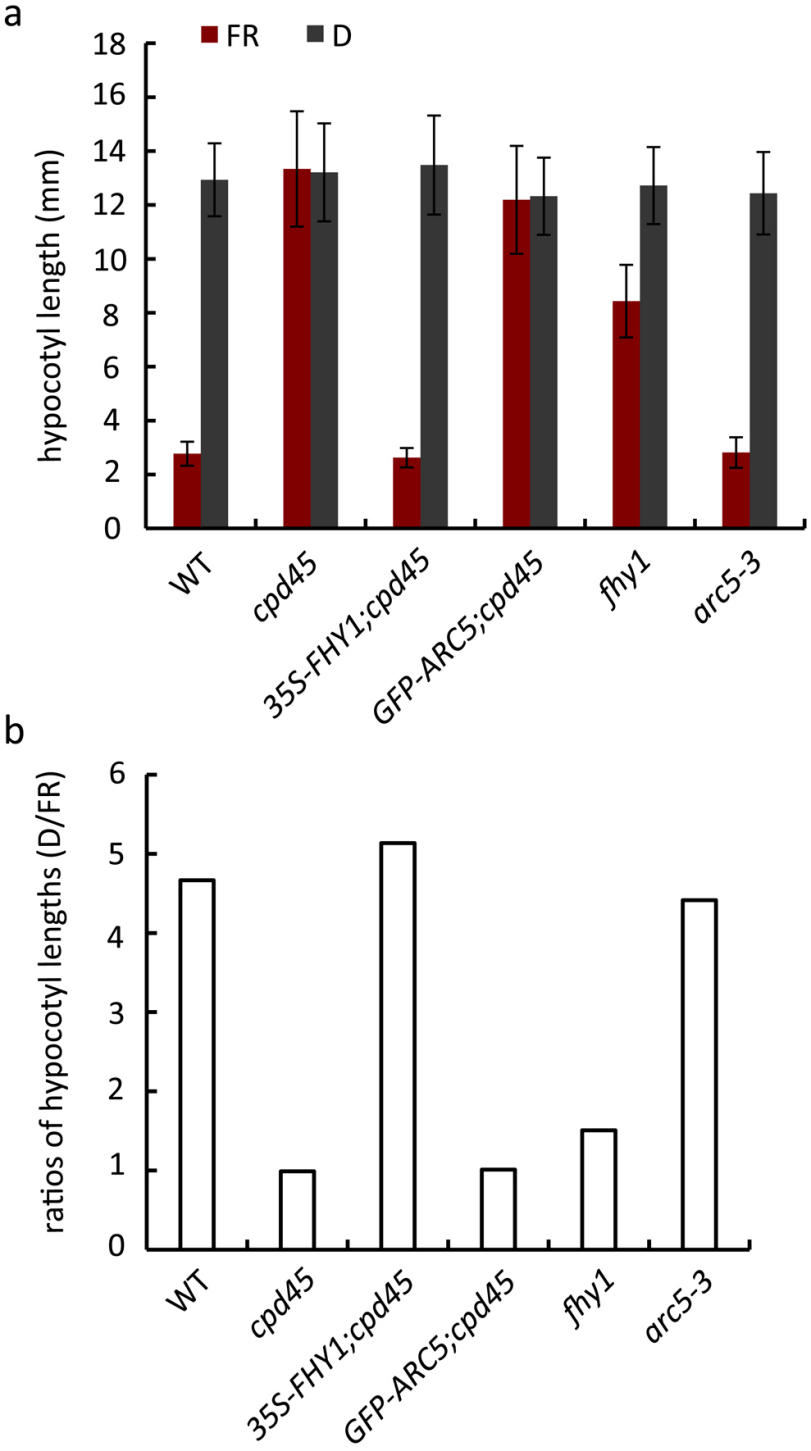
Analysis of hypocotyl length. (a) Hypocotyl length of seedlings of various genetic backgrounds. FR, far-red light (10 μmol·m^−2^·s^−1^); D, dark. n = 30 seedlings. Error bars represent standard deviation. Seeds were plated and hypocotyl lengths were measured after 4 days of incubation under continuous far-red light or in the dark. (b) Corresponding ratios of the hypocotyl length shown in (a).

**Figure 3:**
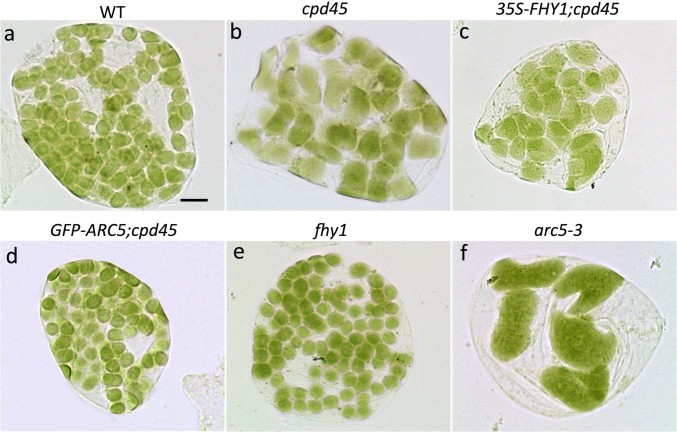
Chloroplast division phenotypes of mesophyll cells with different genetic backgrounds. (a) Wild type. (b) *cpd45* mutant. (c) *cpd45* mutant harboring the *35S-FHY1* transgene. (d) *cpd45* mutant harboring the *35S-GFP-ARC5* transgene. (e) *fhy1* mutant. (f) *arc5-3* mutant. Leaf tissue was sampled from 40-day-old plants. Scale bar represents 10 μm.

**Figure 4:**
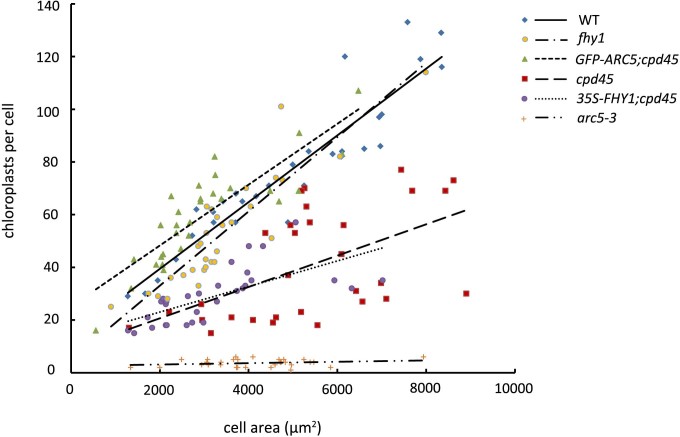
Graph of chloroplast number relative to cell area of 40-day-old plants. The best-fit lines have slopes of 0.0127 (R^2^ = 0.8701), 0.0141 (R^2^ = 0.8069), 0.0115 (R^2^ = 0.7408), 0.0059 (R^2^ = 0.2894), 0.0048 (R^2^ = 0.4459), and 0.0003 (R^2^ = 0.0554) for the wild type (WT), *fhy1*, *GFP-ARC5;cpd45, cpd45*, *35S-FHY1;cpd45*, and *arc5-3*, respectively.

To establish whether the effect of *FHY3/CPD45* on chloroplast division is mediated by *FHY1* and the far-red light signaling pathway, we constitutively overexpressed *35S-FHY1* in the *cpd45* mutant background (*35S-FHY1;cpd45*). After incubating seeds of various genetic backgrounds for four days under far-red light, we measured the hypocotyl lengths of the young seedlings. The hypocotyls of *35S-FHY1;cpd45* transgenic plants (2.625 mm) were significantly shorter than those of the *cpd45* mutant (13.336 mm), but very close to those of the wild type (2.772 mm; Figures 1a, b, c and 2a). However, as observed in the *cpd45* mutant, the chloroplasts of 40-day-old *35S- FHY1;cpd45* plants grown under white light were larger in size and reduced in number relative to the wild type (Figure 3a, b and c). We quantified the severity of the chloroplast phenotype using a best-fit linear plot of chloroplasts per cell versus cell area (Figure 4). The *35S-FHY1;cpd45* plants have a slope of 0.0048, which is similar to that of *cpd45* (0.0059) and very different from that of the wild type (0.0127; Figure 4). These results suggest that the constitutive overexpression of *FHY1* can rescue the far-red light sensing defect of the *cpd45*, but not the chloroplast division defect.

Interestingly, seedlings of *35S-FHY1;cpd45* were stronger than the others both in far-red light or the dark (Figure 1 and S1). Their cotyledon size was approximately twice as large as other plants. We measured the cotyledon size of the seedlings of various genetic backgrounds grown in the dark or far-red light. However, the response to far-red light of the seedlings of *35S-FHY1;cpd45* was similar to that of other plants and the cotyledon size was induced more than two times by far-red light (Figure S1).

### Constitutive overexpression of *ARC5* rescues the chloroplast division defect, but not the far-red light signaling defect, of *cpd45*

To investigate whether the far-red light signaling defect of *cpd45* is caused by the chloroplast division defect, we transformed *35S-GFP-ARC5* into the *cpd45* mutant. After 40 days of growth under white light, we observed the chloroplast division phenotype of leaf mesophyll cells using a microscope. We found that constitutive overexpression of *GFP-ARC5* rescued the chloroplast defects of *cpd45* (Figure 3d), because the chloroplast phenotype of *GFP-ARC5;cpd45* transgenic plants was very similar to that of the wild type. In our best-fit linear plot of chloroplasts per cell against cell area, the slope of *GFP-ARC5;cpd45* (0.0115) was higher than that of *cpd45* (0.0059), and close to that of the wild type (0.0127; Figure 4). In contrast, the hypocotyls of *GFP-ARC5;cpd45* transgenic plants grown under continuous far-red light was 12.190 mm long, more than four times as long as those of the wild type (2.772 mm) and close in length to those of *cpd45* (13.336 mm; Figures 1a, b, d and 2a). Thus, *35S-GFP-ARC5* rescues the chloroplast division defect of *cpd45*, but not the far-red light signaling defect.

**Figure 5:**
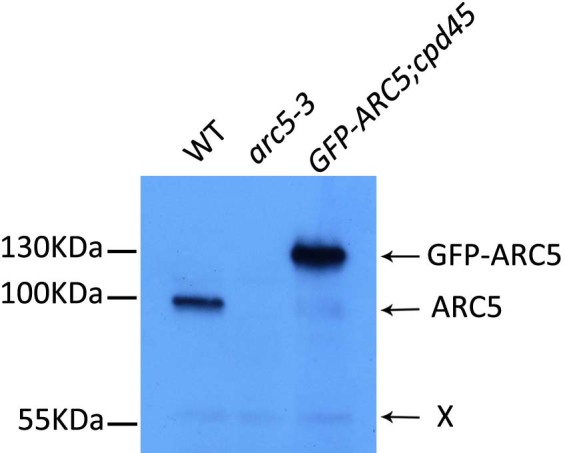
Immunoblot analysis of ARC5 protein levels in wild-type, *arc5-3*, and *GFP-ARC5; cpd45* plants. ARC5 and GFP-ARC5 are indicated, and were approximately 100 kD and 130 kD in size, respectively. X represents a non-specific band, which can be used as a loading control.

We next performed an immunoblot analysis to determine the expression level of the constitutively overexpressed GFP-ARC5 in the *GFP-ARC5;cpd45* transgenic line and to compare this value with that of ARC5 in the wild type (Figure 5). We detected a band of around 100 kD in size in the wild type. This band was absent in *arc5-3* and very faint in *cpd45*, suggesting that it corresponded with ARC5. GFP-ARC5 yielded a band of 130 kD, as expected. Based on our rough estimation, the expression level of GFP-ARC5 was at least five times greater than that of ARC5 in the wild type.

### Mutations in *FHY1* and *ARC5* do not affect the expression of each other

**Figure 6:**
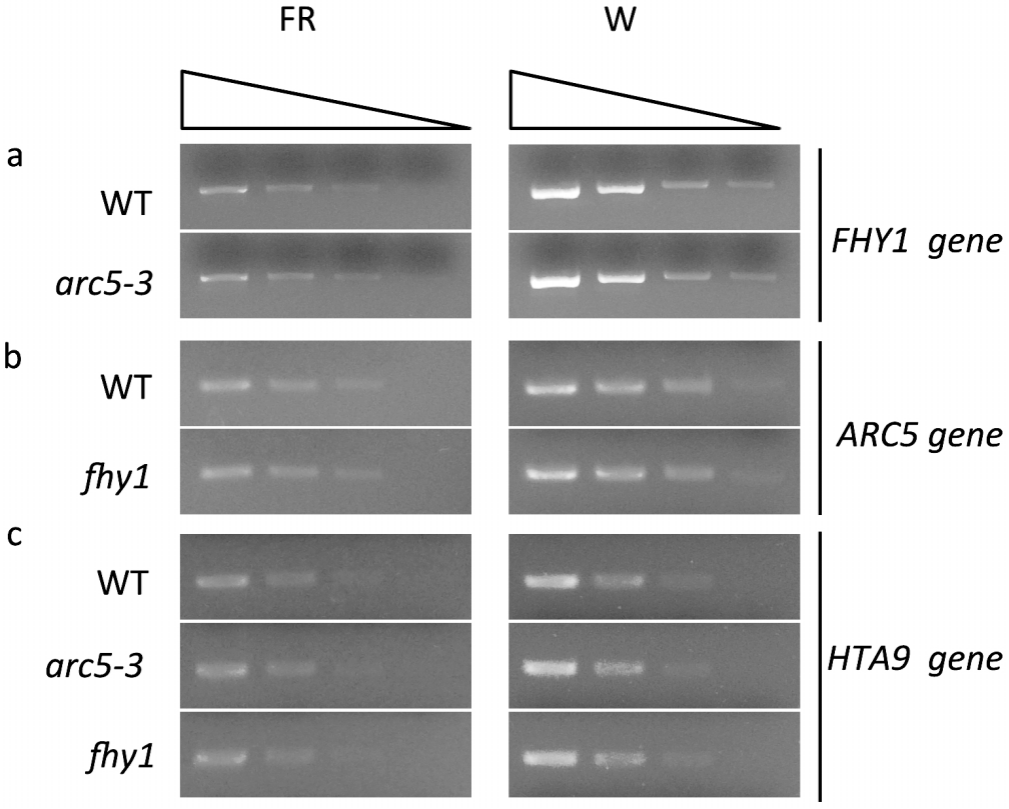
Semi-quantitative PCR analysis of gene expression level in wild type, *arc5-3* and *fhy1* plants. (A) *FHY1* gene. (B) *ARC5* gene. (C) *HTA9* gene (*HISTONE H2A PROTEIN 9*). FR represents plants grown under far-red light for 4 days and W represents plants grown under white light for 40 days. Black triangles indicate that the quantity of cDNA was serially diluted three times with a dilution factor of 4 (from left to right). *HTA9* was used as a control.

We next performed semi-quantitative RT-PCR experiments to further characterize the relationship between *FHY1* and *ARC5* (Figure 6). To avoid saturation of the PCR amplification and for better comparison of the expression levels of the genes, the cDNA used in the experiment was serially diluted three times with a dilution ratio of 4. *HTA9*, which is a constitutively expressed gene, was used as a control. The expression level of *FHY1* in *arc5-3* was similar to that in the wild type, both in four-day-old seedlings grown under far-red light and in the leaves of 40-day-old plants grown under white light. Similarly, the expression level of *ARC5* in *fhy1* was similar to that of the wild type under the above two conditions. The *fhy1* mutant used here (Salk_076131) has a T-DNA insertion in the first intron of *FHY1* and disrupted gene expression (Figure S2). Thus, *FHY1* and *ARC5* do not regulate each other's expression.

### *fhy1* is defective in far-red light signaling but has a normal chloroplast division phenotype

We measured the hypocotyl lengths of *fhy1* seedlings grown under far-red light or in the dark for 4 days and analyzed the chloroplasts of *fhy1* plants grown under white light for 40 days. Our results showed that the hypocotyl length of *fhy1* under far-red light was 8.432 mm, a little more than three times that of the wild type (2.772 mm) (Figures 1a, e and 2). When grown in the dark, the hypocotyl length of *fhy1* was 12.72 mm (Figure 2a). *fhy1* had a chloroplast division phenotype similar to that of the wild type (Figure 3a, e). In the best-fit linear plot of chloroplasts per cell against cell area, the slope of *fhy1* was 0.0141, quite close to that of the wild type (0.0127; Figure 4). This suggests that FHY1 plays an important role in the far-red light signaling pathway, but not in chloroplast division or in the regulation of this process.

### *arc5-3* has an abnormal chloroplast division phenotype and a normal hypocotyl phenotype in far-red light

We measured the hypocotyl lengths and analyzed the chloroplast phenotype of the null allele, *arc5-3*, under the same growth conditions as above. *arc5-3* exhibited a strikingly abnormal chloroplast phenotype, with very large and very few chloroplasts (Figure 3f). The *arc5-3* mutant yielded a slope of 0.0003 in our best-fit linear plot of chloroplasts per cell against cell area (Figure 4), which was much smaller than that of *cpd45* (0.0059) (Figure 4). By contrast, the hypocotyls of the *arc5-3* mutants were of normal length (2.817 mm, versus 2.772 mm in the wild type; Figure 1a, f and Figure 2a). Thus, *ARC5* is an important factor in the process of chloroplast division, but not in the far-red light signaling pathway.

## Discussion

*FHY3/CPD45* is required for the expression of both *FHY1* and *ARC5*^14^,^25^. Previous studies showed that *FHY1* is an important factor in the far-red light signaling pathway^9^,^10^,^15^,^28^. If *FHY3/CPD45* regulated *ARC5* and chloroplast division through *FHY1* and the far-red light signaling pathway, the chloroplast division defect of *cpd45* would be rescued when *FHY1* expression was recovered. However, our results showed that the constitutive expression of *FHY1* only rescued the far-red light signaling defect of *cpd4*5, but not the chloroplast division defect (Figures 1c and 3c). This suggests that *FHY1* does not function in the chloroplast division pathway. Furthermore, our study showed that *fhy1* had elongated hypocotyls under far-red light conditions, but that its mature leaves had a normal chloroplast division phenotype (Figures 1e and 3e), also suggesting that *FHY1* does not function upstream of ARC5. Thus, *FHY3/CPD45* regulates chloroplast division via a pathway that is distinct from far-red light signaling.

Furthermore, it was also unclear whether *FHY3/CPD45* regulated far-red light signaling through the chloroplast division pathway or whether *ARC5* and chloroplast division would affect far-red light signaling. The null allele of *ARC5*, *arc5-3*, exhibited a very severe chloroplast division phenotype (Figures 3f and 4), but its response to far-red light was similar to that of the wild type (Figures 1f and 2). This indicates that *ARC5* is an important factor in chloroplast division, but not in the far-red light signaling pathway. Moreover, a transgene of *GFP-ARC5* driven by the 35S promoter only rescued the chloroplast division defect of *cpd45* (Figure 3d), but not the far-red light signaling defect (Figure 1d). These results suggest that *ARC5* does not function upstream of *FHY1*.

FHY1 has been shown to be required for the nuclear import of phyA and thus the far-red light signaling^29^. As shown in Figures 1 and 2, the *fhy1* mutant had a defect of far-red light sensing like *cpd45*. However, *fhy1* seemed to be not completely blind to far-red light like *cpd45*, because the elongation of its hypocotyls was slightly inhibited by far-red light (Figure 2). FHL had been shown to have some redundancy with FHY1 for the nuclear import of phyA, but with a minor role^10^,^12^. So, a small part of phyA could be imported into the nucleus with the assistance of FHL for far-red light sensing. Alternatively but unlikely, the T-DNA in *fhy1* was inserted into an intron of *FHY1* gene (Figure S2), so that there might be still a very small amount of correctly spliced mRNA.

Seedlings of *35S-FHY1;cpd45* appeared to be stronger than the others both in far-red light or the dark (Figure 1 and S1). Especially, the cotyledon size appeared to be larger. 35S promoter is a constitutive and very strong promoter. It could cause an overexpression of *FHY1*. This might increase the nuclear import efficiency of phyA or other proteins and affect the development of plants.

In contrast to the complete absence of ARC5 in *arc5-3*, our immunoblot analysis suggested that there was still a low level of ARC5 in *cpd45* (Figure 5). This is probably because the EMS-induced point mutation in *cpd45* only caused an amino acid substitution in the encoded protein, resulting in a leaky mutation^25^. Therefore, there is still some trace activity of FHY3/CPD45 and a very slight activation of *ARC5* expression in *cpd45*. Alternatively, *cpd45* may have totally lost its activity and the *ARC5* expression may be due only to basal expression. The lack of ARC5 in *arc5-3* could be due to the incorrect splicing of the pre-mRNA, which introduced a premature stop codon^25^. Regardless, the low level, but not the absence, of ARC5 in *cpd45* can explain why its chloroplast division defect is not as severe as that of *arc5-3*.

Our results also suggest that the constitutive overexpression of GFP-ARC5 can rescue the chloroplast division defect in *cpd45* (Figure 3d). The level GFP-ARC5 in the overexpressing plants is at least five times greater than the level of ARC5 in the wild type (Figure 5). However, previous studies have shown that overexpression of a chloroplast division protein, such as FtsZ, MinD, or MinE, could inhibit chloroplast division^30^,^31^,^32^. This is probably because these proteins form a complex with a stringent stoichiometry and overexpression of one component causes misassembly of the complex and then faulty chloroplast division. The working mechanism of ARC5 may be different from that of other chloroplast division proteins.

Our gene expression analysis showed that expression of *FHY1* or *ARC5* was not substantially affected in the alternative mutant under both white light and far-red light conditions (Figure 6). This further suggests that these two genes do not function in the same pathway, i.e., that they do not function upstream or downstream of each other. Instead, they are both regulated by *FHY3/CPD45* in parallel.

Many lines of evidence suggest that *FHY3/CPD45* regulates circadian rhythm in *Arabidopsis*^33^,^34^,^35^. For instance, *FHY3/CPD45* regulates the expression of *ELF4* (*Early Flower 4*) and plays a dominant role in the *CCA1*/*LHY-TOC1* circadian clock feedback circuit^36^,^37^, by binding to the *ELF4* promoter, and thereby regulates the biological clock of plants^35^.

**Figure 7:**
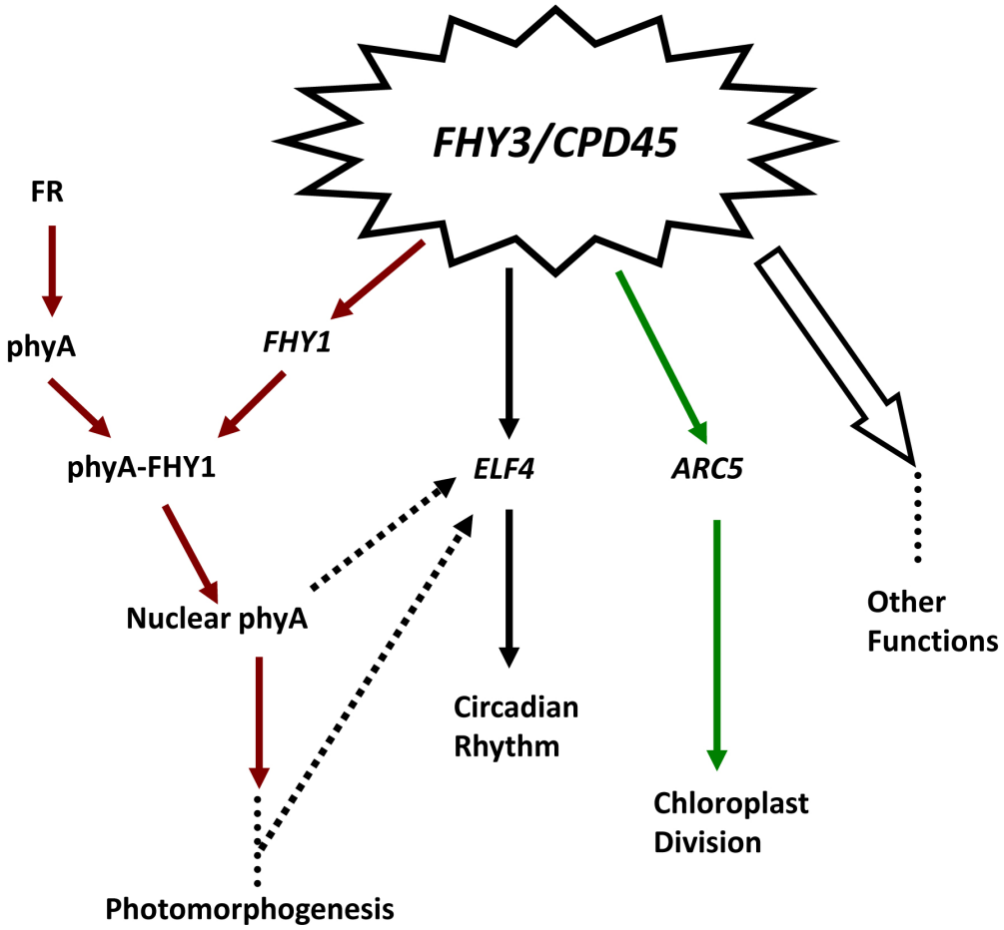
A simplified genetic model of *FHY3*/*CPD45*-mediated gene regulatory pathways. *CPD45* (also known as *FHY3*) regulates the expression of many genes and is an important regulator of chloroplast division, the far red-light signaling pathway, circadian rhythm, and other biological processes. Arrows indicate genes that are directly regulated by *FHY3/CPD45* and the pathways in which they are involved, dotted arrows indicate possible pathways that remain to be confirmed, and dotted lines represent unknown components or factors and steps not indicated.

Collectively, the information presented in this and previous studies suggest that *FHY3*/*CPD45* is a crunode in the gene regulatory network, which links pathways of photomorphogenesis, circadian rhythm, chloroplast division and others. Therefore, we propose a genetic model of *FHY3/CPD45*-mediated gene regulatory pathways (Figure 7). In this model, *FHY3/CPD45* is an important regulator of many genes that controls chloroplast division, far-red light signaling, and the circadian rhythm through regulating the expression of *ARC5*, *FHY1*, and *ELF4*, respectively. As an important transcription factor, FHY3/CPD45 may also regulate the expression of many other genes and govern many other biological pathways.

This work has finally clarified that the relationship between *FHY1* and *ARC5* in the gene regulatory network is parallel. It is well known that light signals are essential regulators of circadian rhythm and many other biological processes. Therefore, future studies should investigate the relationship between the far-red light signaling pathway and circadian rhythm and other pathways. Such studies may reveal a complex gene regulatory network mediated by *FHY3/CPD45*.

## Methods

### Plant materials

All *Arabidopsis thaliana* plants used in this study were in the Columbia-0 ecotype background. *arc5-3* is a mutant allele of *ARC5*, and *cpd45* is a mutant allele of *FHY3*^25^. *fhy1* (Salk_076131) is a T-DNA insertion mutant of *FHY1*.

### Growth conditions

Sterilized seeds were plated on 1/2 MS medium with 1% sucrose and 0.8% agar. After stratification at 4°C for 2 days, the plates were placed vertically under continuous far-red light (720 ~ 740 nm, 10 μmol·m^−2^·s^−1^) or in the dark for 4 days without photoperiod at 22°C as before Ref. 25. Soil-grown plants were grown from seed in a growth chamber under white light (90 ~ 120 μmol·m^−2^·s^−1^) with 40 ~ 60% relative humidity at 22°C and a photoperiod of 16-h-light/8-h-dark.

### Measurement of hypocotyl length and cotyledon size

Plates were removed from the far-red light or dark box after 4 days of growth. Images were first taken with a dissection microscope and a USB2.0 digital camera (Changheng, Beijing) and then the hypocotyl length and cotyledon size (area) were measured with the software Image Analysis System 10.0 (Changheng, http://www.crisoptical.com/lm2_41_344.htm). The sample size was 30.

### Leaf fixation and chloroplast phenotype analysis

To characterize the chloroplast phenotype, leaf tissues from 40-day-old plants were fixed in an Eppendorf tube with 3.5% glutaraldehyde for 1 h in darkness, the glutaraldehyde was then replaced with 0.1 M Na_2_EDTA (PH = 9.0), and the tube was placed in a water bath of 55°C for 2 h. Images were taken with a USB2.0 digital camera (Changheng, Beijing) coupled to an Olympus CX21 microscope (Olympus, Tokyo). The chloroplast division phenotype was quantified as described previously^25^. Thirty mesophyll cells of each group were used for the quantification.

### Identification of an *fhy1* T-DNA insertion mutant, Salk_076131

The *fhy1* T-DNA insertion mutant Salk_076131 was obtained from the Arabidopsis Biological Resource Center. The sequence flanking the T-DNA insertion site was amplified using primers LBa1 (5'- TGG TTC ACG TAG TGG GCC ATC G -3') and RP (5'- TGG TAG GCT TCT TTG TCT CAT G -3') and sequenced. The T-DNA insertion site was then verified by sequence analysis.

### Plasmid construction

*FHY1* were amplified from the genomic DNA of wild-type plants using primers 5'- GCG ATC TAT GCC TGA AGT GG -3' and 5'- CGA CCA TGG GAT ACT CTT GAA CAC AAG ATT GG -3' and then inserted into 3302Y3 between the StuI and NcoI cutting sites to generate *35S-FHY1*. 3*5S*-*GFP-ARC5* was constructed in a previous study^25^. To obtain the pET30a-*ARC5* expression vector, *ARC5* was amplified from the wild-type cDNA with the primers 5'- GGA GGA TCC GAG CGG TGG AGT CTT TAC G -3' and 5'- CCA CTC GAG AAA GCT CAG GAT CAA CTT GCA TCA C -3'. The PCR product was cloned into the pET30a vector between the XhoI and XbaI sites.

### RNA extraction and semi-quantitative RT-PCR analysis

RNA was isolated from seedlings grown on plates under far-red light for 4 days or from the leaves of 40-day-old plants grown in soil under white light, using an RNApure Total RNA Isolation Kit (Aidlab, Beijing) and reverse transcribed with a RevertAid First Strand cDNA Synthesis Kit (Thermo Fisher). Semi-quantitative RT-PCR analysis was performed as before Ref. 38. The cDNA templates used for PCR were serially diluted three times at a dilution ratio of 4. *FHY1* was amplified with the primers 5'- GCG ATC TAT GCC TGA AGT GG -3' and 5'- GAG TAG AAT CAT CTT GGT TAA CAG TCC -3'. *ARC5* was amplified with the primers 5'- GCT AAT ACC GAA TGC AGG GAT G -3' and 5'- GCC TTC GCA ACT GCT ATA ACA C -3'.

### Protein purification, antibody preparation and immuno-blot analysis

The pET30a-*ARC5* plasmid was isolated and transformed into the Rosetta strain of *E. coli* for protein expression. Bacterial cultures were incubated at 37°C until the OD_600_ reached 0.4–0.6. Then, 1 mM IPTG(isopropyl β-D-thiogalactopyranoside) was added to the culture to induce the expression of *ARC5*. Bacterial cells were lysed by ultra-sonication and the induced ARC5 protein was purified using Ni Sepharose 6 Fast Flow (GE Healthcare). The polyclonal and monoclonal antibodies against ARC5 were raised in mouse. For immuno-blot analysis, proteins were separated by SDS-PAGE and transferred to Immun-Blot PVDF membrane (Bio-Rad). Blots were probed with anti-ARC5 mouse monoclonal antibodies at a dilution of 1:1000 for 1 h. Secondary antibodies of the Goat anti-Mouse IgG-conjugated HRP (1:10000 dilution) and an eECL Western Blot Kit (Beijing ComWin Biotech Company) were then used.

## Author Contributions

N.C., Y.G. and L.Z. performed the experiments. N.C., Y.G. and X.L. prepared the manuscript. H.G. provided advice on the experiments and improved the manuscript.

## Supplementary Material

Supplementary Information Supplementary Information
