# Supplementary material for: Arabidopsis FHY3/CPD45 regulates far-red light signaling and chloroplast division in parallel
Source: Sci Rep. 2015 Apr 15;5:9612. doi: 10.1038/srep09612 (PMC4397536; doi:10.1038/srep09612)
Supplement: Supplementary Information [file srep09612-s1.doc]

***Arabidopsis FHY3/CPD45* regulates far-red light signaling and chloroplast division in parallel**

Ning Chang^*^, Yuefang Gao^*^, Lin Zhao, Xiaomin Liu and Hongbo Gao

College of Biological Sciences and Biotechnology, Beijing Forestry University, Beijing, 100083, China


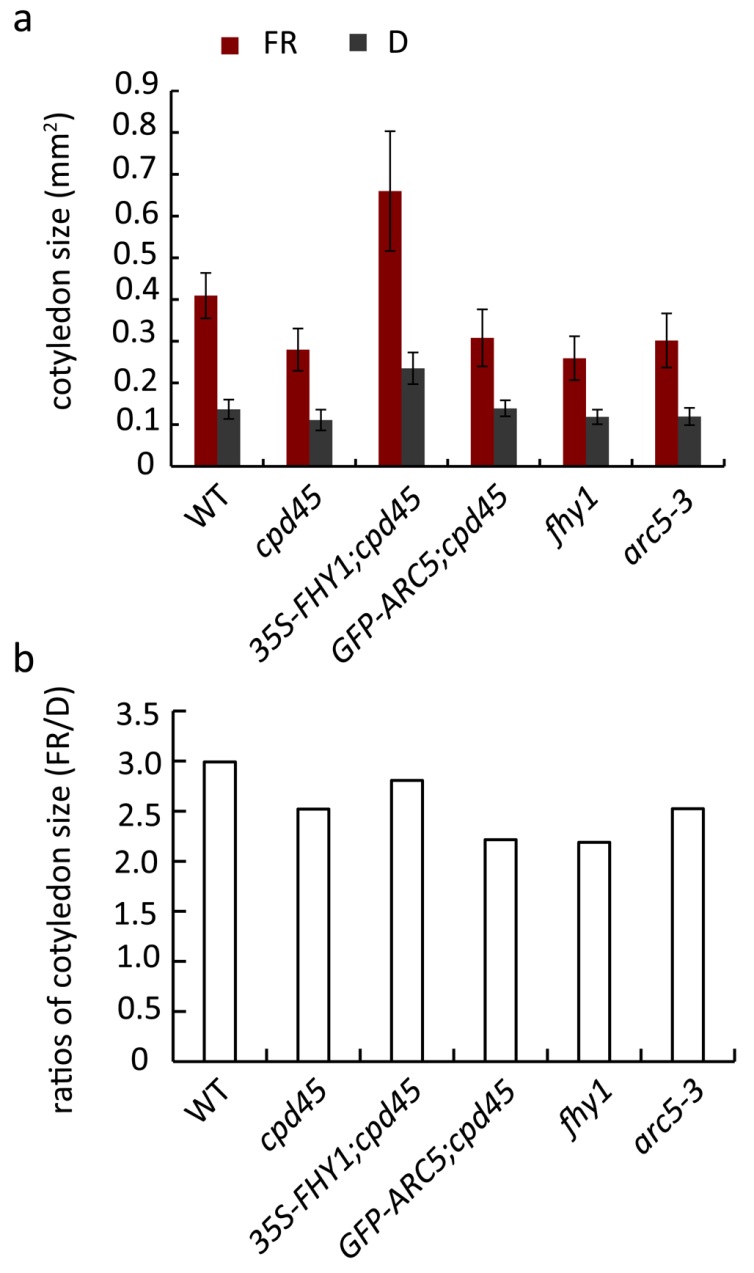


**Figure S1 | Analysis of cotyledon size.** (a) Cotyledon size of seedlings of various genetic backgrounds. FR, far-red light (10 μmol·m^-2^·s^-1^); D, dark. n=30 seedlings. Error bars represent standard deviation. Seeds were plated and cotyledon sizes were measured after 4 days of incubation under continuous far-red light or in the dark. (b) Corresponding ratios of the cotyledon size shown in (a).


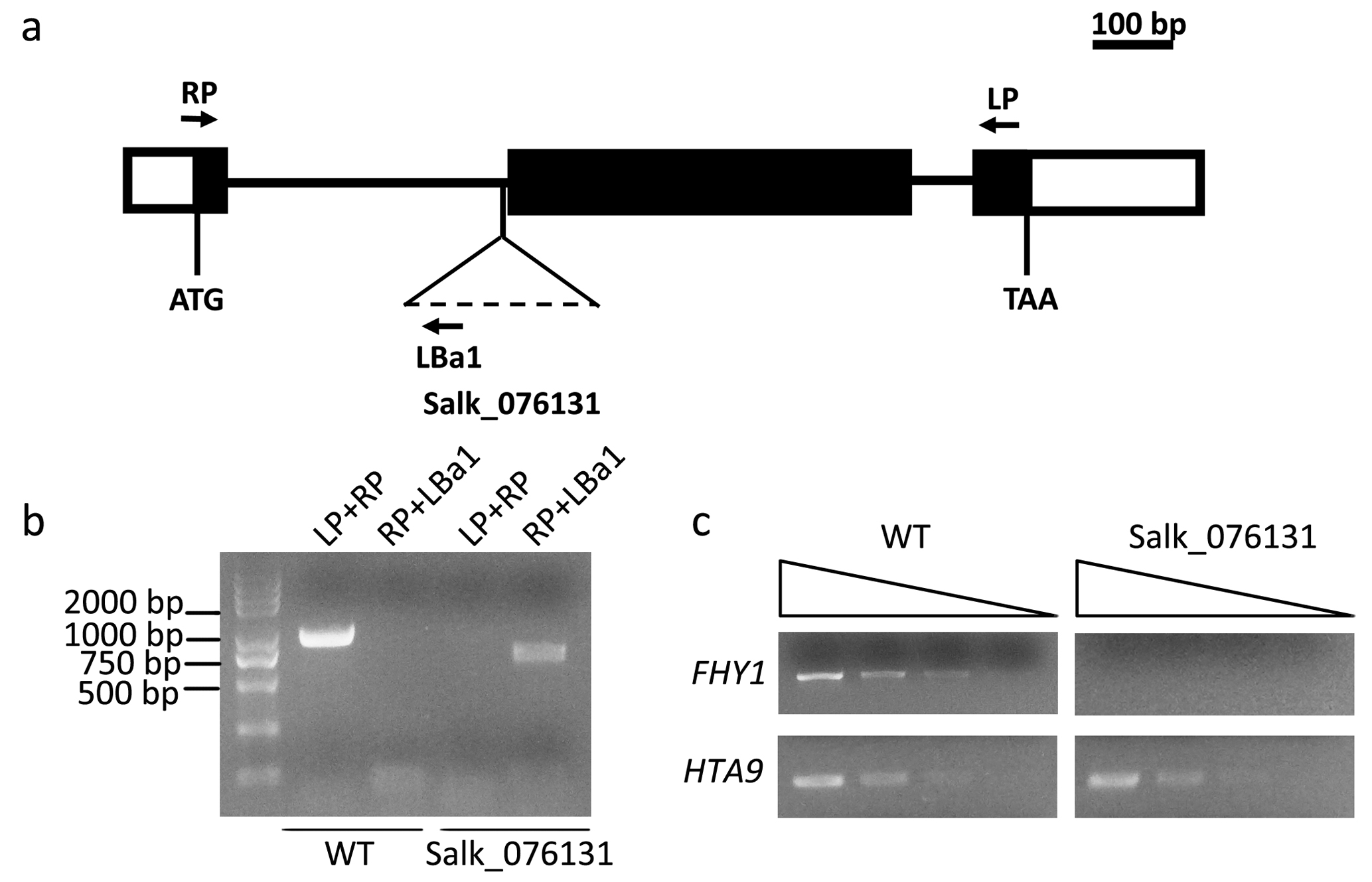


**Figure S2 | Identification of a T-DNA insertion mutant of *FHY1*.** (a) Gene structure of *FHY1*. Boxes represent exons (white boxes, 5’- and 3’- untranslated regions; black boxes, protein coding sequence); solid lines represent introns. ATG is the start codon and TAA is the stop codon. The position of the T-DNA and the position and direction of primers LP, LBa1, and RP are indicated. (b) PCR verification of the T-DNA insertion mutant, Salk_076131. Both the wild-type and mutant plants were verified with primer pairs LP+RP and RP+LBa1. The PCR product amplified with primers LP and RP is 971 bp and with primers RP and LBa1 is 717 bp. The left lane is D2000 Plus DNA ladder. (c) Semi-quantitative PCR was conducted to investigate the expression level of *FHY1* in Salk_076131 and the wild-type seedlings grown in far-red light. *HTA9* was used as a control. The PCR template was serially diluted three times from left to right with a dilution ratio of 4.
